# Intelligence in youth and health behaviours in middle age

**DOI:** 10.1016/j.intell.2018.04.005

**Published:** 2018

**Authors:** Christina Wraw, Geoff Der, Catharine R. Gale, Ian J. Deary

**Affiliations:** a Centre for Cognitive Ageing and Cognitive Epidemiology, Department of Psychology, University of Edinburgh, 7 George Square, Edinburgh, Scotland EH8 9JZ, UK; b MRC/CSO Social & Public Health Sciences Unit, 200 Renfield Street, University of Glasgow, Glasgow G2 3QB, UK; c MRC Lifecourse Epidemiology Unit, University of Southampton, Southampton General Hospital, Southampton SO16 6YD, UK

## Abstract

**Objective:**

We investigated the association between intelligence in youth and a range of health-related behaviours in middle age.

**Method:**

Participants were the 5347 men and women who responded to the National Longitudinal Survey of Youth 1979 (NLSY-79) 2012 survey. IQ was recorded with the Armed Forces Qualification Test (AFQT) when participants were aged 15 to 23 years of age. Self-reports on exercise (moderate activity, vigorous activity, and strength training), dietary, smoking, drinking, and oral health behaviours were recorded when participants were in middle age (mean age = 51.7 years). A series of regression analyses tested for an association between IQ in youth and the different health related behaviours in middle age, while adjusting for childhood socio-economic status (SES) and adult SES.

**Results:**

Higher IQ in youth was significantly associated with the following behaviours that are beneficial to health: being more likely to be able to do moderate cardiovascular activity (Odds Ratio, 95% CI) (1.72, 1.35 to 2.20, *p* < .001) and strength training (1.61, 1.37 to 1.90, *p* < .001); being less likely to have had a sugary drink in the previous week (0.75, 0.71 to 0.80, *p* < .001); a lower likelihood of drinking alcohol heavily (0.67, 0.61 to 0.74, *p* < .001); being less likely to smoke (0.60, 0.56 to 0.65, p < .001); being more likely to floss (1.47, 1.35 to 1.59, *p* < .001); and being more likely to say they “often” read the nutritional information (1.20, 1.09 to 1.31, *p* < .001) and ingredients (1.24, 1.12 to 1.36, p < .001) on food packaging compared to always reading them. Higher IQ was also linked with dietary behaviours that may or may not be linked with poorer health outcomes (i.e. being more likely to have skipped a meal (1.10, 1.03 to 1.17, *p* = .005) and snacked between meals (1.37, 1.26 to 1.50, *p* < .001) in the previous week). An inverted u-shaped association was also found between IQ and the number of meals skipped per week. Higher IQ was also linked with behaviours that are known to be linked with poorer health (i.e. a higher likelihood of drinking alcohol compared to being abstinent from drinking alcohol (1.58, 1.47 to 1.69, *p* < .001)). A u-shaped association was found between IQ and the amount of alcohol consumed per week and an inverted u-shaped association was found between IQ and the number of cigarettes smoked a day. Across all outcomes, adjusting for childhood SES tended to attenuate the estimated effect size only slightly. Adjusting for adult SES led to more marked attenuation but statistical significance was maintained in most cases.

**Conclusion:**

In the present study, a higher IQ in adolescence was associated with a number of healthier behaviours in middle age. In contrast to these results, a few associations were also identified between higher intelligence and behaviours that may or may not be linked with poor health (i.e. skipping meals and snacking between meals) and with behaviours that are known to be linked with poor health (i.e. drinking alcohol and the number of cigarettes smoked). To explore mechanisms of association, future studies could test for a range of health behaviours as potential mediators between IQ and morbidity or mortality in later life.

## Highlights

• Links between intelligence in youth and mid-life health behaviours were examined.
• Higher IQ was associated with a range of healthier behaviours in mid-life.
• There were non-linear associations between IQ and unhealthy behaviours.
• There was essentially no attenuation after adjusting for childhood SES.
• Statistical significance was largely maintained after adjusting for adult SES.

Intelligence measured in youth is associated with lower rates of premature all-cause and cause-specific mortality (Batty, Deary, & Gottfredson, 2007; Calvin et al., 2011; Calvin et al., 2017; Deary, Whiteman, Starr, Whalley, & Fox, 2004; Whalley & Deary, 2001) and a number of leading chronic health conditions including, but not limited to, cardio-vascular disease (CVD), diabetes, chronic lung disease, and high blood pressure (Der, Batty, & Deary, 2009; Wraw, Deary, Gale, & Der, 2015). It is possible that health behaviours lie on the pathways that link early life intelligence with morbidity and mortality in middle and later life (Gottfredson & Deary, 2004). The aim of the present study is to investigate the association between intelligence in youth and a range of health-related behaviours in middle age. The behaviours of interest in the present study are exercise habits, diet, smoking, drinking, and oral hygiene behaviours. We will cover the literature on the link between IQ and each of these behaviours separately.

Many of the studies that have examined the associations between physical activity in adulthood and early-life cognitive ability have found higher intelligence to be associated with being more physically active (Batty, Deary, & Macintyre, 2006; Clouston, Richards, Cadar, & Hofer, 2015; O'Callaghan, Bor, & Najman, 2012; Osler, Godtfredsen, & Prescott, 2008). One study that was based on the Mater-University of Queensland Study of Pregnancy (*n* = 7223) found a mixture of results upon investigating the associations between intelligence and different measures of physical activity (O'Callaghan et al., 2012). They found that lower IQ scores at age 21 years were associated with doing less moderate activity over the past 2 weeks and 6 months at the age of 21 years. This study also found an inverted u-shaped association between IQ and both vigorous exercise and vigorous activity (apart from exercise) over the past two weeks. For example, with the number of vigorous activity sessions over the past two weeks broken into four categories (i.e. Nil, 1 to 3, 4 to 9, and 10 +) those who did either no sessions (IQ 102.9) or >10 sessions (IQ 102.8) had lower scores on the Peabody verbal IQ test than those who reported doing either 1 to 3 (IQ 104.9) or 4 to 9 (IQ 104.4) sessions (F = 5.45, *p* = .001). As this was a cross sectional study, the direction of the association could not be determined. Studies have also identified null associations between early-life IQ and whether or not people exercised regularly (Modig & Bergman, 2012) and with the frequency of physical activity (Batty, Deary, & Macintyre, 2006; O'Callaghan et al., 2012). The longitudinal studies cited are limited because they only included measures of whether or not people exercise and the frequency of exercise (Batty, Deary, & Macintyre, 2006; Clouston et al., 2015; Osler et al., 2008). No data were collected on the minutes of exercise taken each week in any of the cited studies which is needed in order to establish whether people are as active as the US guidelines recommend in the 2008 Physical Activity Guidelines for Americans (U.S Department of Health and Human Services, 2008. The present study aims to shed light on this area of research by exploring the association between early-life intelligence and both the ability to engage in cardiovascular activity and the amount of cardiovascular activity per week at middle age. Cardiovascular activities are activities that increase the heart rate and breathing rate to increase the delivery of oxygen to working muscles. This will be the first study that we are aware of that examines the association between early life intelligence and strength training.

The few studies that have investigated the association between early-life cognitive ability and diet-related behaviours in adulthood found higher IQ to be associated with healthier dietary choices (Batty, Deary, & Macintyre, 2006; Crichton et al., 2015; Kanazawa, 2013; Osler et al., 2008). One study found null associations between intelligence and three dietary habits (Lyerly & Reeve, 2014). Lyerly and Reeve (2014) studied the relationship between intelligence and dietary behaviours in a sample of 4078 men and women using data from the 2010 NLSY-79 survey. They found no association between intelligence in youth and three measures of dietary habits (frequency with which participants consumed fast food and sugary drinks and how often they skipped meals in the past 7 days). However, their results are questionable due to the methodological limitations of their study design. Their measures of dietary behaviours were positively skewed and they used ordinary least squares regression to analyse their data, which is not the best model to use on skewed measures. The present study investigates the same associations using a generalized linear regression model, which is more appropriate for skewed measures, on data from the NLSY79 2012 survey. The association between intelligence and an additional dietary behaviour (snacking between meals) and two measures of reading food labels will also be included in the present study.

Studies that examined the association between early-life intelligence and alcohol consumption in middle age have found mixed results. Some studies have found that more intelligent people are less likely to abstain from drinking and are more likely to have drinking problems, drink more frequently, and to have a higher intake of alcohol in adulthood (Osler et al., 2008; Batty et al., 2008; Clouston et al., 2015; Cheng & Furnham, 2013. Clouston et al. (2015) found higher intelligence in adolescence was associated with heavy drinking (i.e. men having >3 drinks on one occasion and women > 2 drinks on one occasion) at around 50 years of age in the Medical Research Council's National Survey of Health and Development, the combined OR for men and women was 1.34 (95% C.I 1.10 to 1.62); null associations were found in the Wisconsin Longitudinal Study and the National Child Development Study. Other studies have found that higher early-life intelligence is protective against some of the more risky drinking behaviours, such as the likelihood of having hangovers (Batty, Deary, & Macintyre, 2006), binge drinking (Cheng & Furnham, 2013), being a heavy drinker, and being admitted to hospital for or dying from drinking-related causes (Sjölund, Allebeck, & Hemmingsson, 2011; Sjölund, Hemmingsson, & Allebeck, 2015; Sjölund, Hemmingsson, Gustafsson, & Allebeck, 2015). For example, Batty, Deary, & Macintyre (2006) analysed data from the Aberdeen children of the 1950s study (*n* = 5340) and found a SD increment in IQ at age 11 was associated with lower odds of having a hangover in middle age (OR 0.80, 95% CI 0.72 to 0.89). Cheng and Furnham (2013) analysed data from the National Childhood Development Study 1958 (*n* = 6478) and found a SD increment in IQ at age 11 was linked with reduced odds of binge drinking at age 50 years (OR 0.76, 95% CI 0.70 to 0.83). The previous mixed results might suggest a non-linear relationship that might be better captured by a continuous measure, which was not included in the cited studies. One of the aims of the present study is to clarify the nature of the association between early-life intelligence and drinking behaviours by exploring the link between IQ and abstaining from alcohol, amount of alcohol consumed as a continuous measure, and binge drinking behaviours in middle age.

A number of studies have found higher early-life intelligence to be related to smoking in middle age (Batty, Deary, & Macintyre, 2007; Daly & Egan, 2017; Hart et al., 2003; Modig & Bergman, 2012; Osler et al., 2008; Clouston et al., 2015; Hemmingsson, Kriebel, Melin, Allebeck, & Lundberg, 2008). For example, Osler et al. (2008) analysed data from the Metropolit 1953 Danish male birth cohort study (*n* = 6292) and found a SD increment in IQ at age 18 was linked with reduced odds of being a smoker (OR 0.67, 95% CI 0.63 to 0.70) and increased odds of being an ex-smoker (OR 1.38, 95% CI 1.28 to 1.48) at 51 years of age. Studies in this area tend to look at the association between IQ and smoking status (current smoker, ex- smoker, never smoker) rather than the quantity of cigarettes smoked. Two studies have analysed the latter (Taylor et al., 2005; Wennerstad et al., 2010). Using structural equation modelling, Taylor et al. (2005) found a link between lower IQ and a higher consumption of cigarettes, which was fully mediated by adult social deprivation. The study by Wennerstad et al. (2010) was based on the 29,524 Swedish male twin conscripts. Data about smoking were collected through two separate surveys: the Screening Across the Lifespan Twin Study (SALT) and the Study of Twin Adults: Genes and Environment (STAGE). SALT and STAGE are both surveys of monozygotic (MZ) and dizygotic (DZ) twins that were born in Sweden, which were used to screen for common diseases. The twins in SALT were born before 1958 and the twins in STAGE were born between 1959 and 1985. Wennerstad et al. (2010) found that, with higher IQ scores, the number of cigarettes smoked per day was lower in the STAGE survey (*r* = −0.20 *p* < .001), but they did not find an association in the SALT survey. The present study expands on this area of research by investigating the association between early life intelligence and both current smoking status and, for those who smoke, number of cigarettes smoked per day at age 50.

The present authors are aware of two studies that analysed the association between intelligence and oral health (Der et al., 2009; Sabbah & Sheiham, 2010). Higher intelligence was linked with better oral health outcomes in both studies (Der et al., 2009; Sabbah & Sheiham, 2010). Der et al. (2009) analysed data from the National Longitudinal Survey of Youth 1979 (*n* = 7476) and found a SD increment in IQ in youth was linked with reduced odds of having tooth and gum problems at 40 years of age (OR 0.73, 95% CI 0.66 to 0.81). Sabbah and Sheiham (2010) analysed cross sectional data on 20 to 59 year olds (*n* = 4390) from the Third National Health and Nutrition Examination Survey and found that lower score on the two tests of intelligence (the Symbol Digit Substitution Test and the Serial Digit Learning Test) were linked with poorer oral health. For example, lower scores on the Symbol Digit Substitution Test was linked with being more likely to have missing tooth surface (rate ratio 1.52, 95% CI 1.31–1.77) and decayed tooth surface (rate ratio 1.91, 95% CI 1.53–2.38). However, the study by Sabbah and Sheiham (2010) was cross-sectional and analysed measures of intelligence and oral health status that were taken around the same time so the direction of the association cannot be determined. No previous study has looked at the oral health behaviours of brushing and flossing teeth. The present study is the first to test the longitudinal relationship between intelligence in youth and these two oral hygiene habits in middle age.

Many of the previous studies that have investigated the longitudinal association between early-life intelligence and health-related behaviours are limited because their investigations only included a single (Daly & Egan, 2017; Sjölund et al., 2011; Sjölund, Hemmingsson, Gustafsson, & Allebeck, 2015; Taylor et al., 2003; Wennerstad et al., 2010) or a few health behaviours (Batty, Deary, & Macintyre, 2006; Batty, Deary, Schoon, & Gale, 2006; Clouston et al., 2015; Modig & Bergman, 2012). Also, as discussed above, the results are often mixed. The present study will help to contribute to this relatively new area of research within the field of cognitive epidemiology, and will examine the links between intelligence in youth and thirteen different health behaviours that are linked with chronic disease, using the NLSY-79 cohort. Lifestyles and health habits tend to be well established and predictive of health outcomes by midlife (Myint, Smith, Luben, & Surtees, 2011) so the present study will analyse the association between IQ in youth and health habits in middle-aged adults.

## 1 Methods

### 1.1 Sample

The National Longitudinal Survey of Youth 1979 (NLSY-79) is an on-going longitudinal study. The initial sample was representative of non-institutionalized young people who lived in the United States and involved 12,686 participants aged 14–21 years on 31st of December 1978. Socially and economically disadvantaged non-Black and non-Hispanic people as well as Black and Hispanic ethnic groups were oversampled. The NLSY-79 study has been described in detail elsewhere (Bureau of Labor Statistics, 2012). The present analyses were based on the 5347 men and women who responded to the 2012 survey and had complete information on AFQT, sex, age at interview, ethnicity, childhood SES, and adult SES.

### 1.2 Measures

#### 1.2.1 Intelligence

Intelligence was measured with the Armed Forces Qualification Test (AFQT), 1989 re-normed version. Respondents were aged 15–23 years when they sat this test in 1980. The AFQT is a sub-component of the Armed Services Vocational Aptitude Battery and is made-up of the four following subtests: Arithmetic reasoning (30 items), mathematics knowledge (25 items), word knowledge (35 items), and paragraph comprehension (15 items). The time limit for the test was 84 min (Maier, 1993). AFQT is predictive of academic and job performance (Welsh, Kucinkas, & Curran, 1990). The AFQT variable used in the present study was downloaded from The Bell Curve Page (Herrnstein & Murray, 1994). This measure was scored as a percentile, and was then z-scored.

### 1.3 Health behaviours

#### 1.3.1 Physical activity habits

Participants were asked if they were able to engage in moderate cardiovascular activity, vigorous cardiovascular activity, and/or strength training. For participants who said they could engage in each respective activity, information was collected on the typical number of minutes spent doing each cardiovascular activity and the typical number of strength training sessions they did each week. All three of these measures were categorised because their distributions were heavily skewed. We chose cut-points for the categorisations based on the 2008 Physical Activity Guidelines for Americans, for cardiovascular activity and cut-points based on research recommendations, for strength training (Candow & Burke, 2007; O'Keefe et al., 2012; U.S Department of Health and Human Services, 2008; Wen et al., 2011; Wernbom, Augustsson, & Thome, 2007).

The measure of minutes of moderate activity per week was broken into the following categories: inactive (<75 min), a little (75 to 149 min), a medium amount (150 to 509 min), a lot (510 min or more). As guidelines recommend doing 150 min of moderate cardiovascular activity each week, *a little* was selected as the reference category because this group took some exercise but slightly less than the recommended amount (U.S Department of Health and Human Services, 2008; Wen et al., 2011). The measures of moderate and vigorous activity were kept as separate measures and were not combined for the analyses; however, 19% (*n* = 1215) of the respondents to the question on vigorous activity provided the same answer to both vigorous and moderate activity.

The measure of minutes of vigorous activity per week was broken into the following categories: inactive (<38 min), a little (38 to 74 min), a medium amount (75 to 254 min), and a lot (255 min of more). As the guidelines recommend doing 75 min of vigorous cardiovascular activity each week, *a little* was selected as the reference category because this group took some exercise but slightly less than the recommended amount (U.S Department of Health and Human Services, 2008; Wen et al., 2011).

The measure of strength training in sessions per week was broken into the following categories: inactive (zero sessions), a medium amount (1 to 3 sessions), a lot (4 or more sessions). As guidelines recommend doing two or more resistance training sessions per week and research recommends doing 1 to 3 sessions per week, *a medium amount* was used as the reference category. Research considers doing 4 or more sessions per week to be a high-frequency of resistance training (Candow & Burke, 2007; Wernbom et al., 2007).

#### 1.3.2 Diet and nutritional habits

Participants were asked to indicate how frequently (always, often, sometimes, rarely or never) they read nutritional information and ingredients when they buy a food item for the first time. Information was also collected on how frequently participants ate fast food, snacked between meals, skipped a meal, and had a sugary drink in the past 7 days.

#### 1.3.3 Smoking habits

Participants were asked if they currently smoke cigarettes. The participants who reported that they did smoke were asked how many cigarettes they smoked a day.

#### 1.3.4 Drinking habits

Participants were asked if they had drunk alcohol in the past 30 days. For those who said they had drunk alcohol, information was collected on the number of days they had drunk in the last 30 days and the number of drinks they tended to have when they drank. These data were used to construct a continuous variable that measured the number of drinks participants had per week. Participants were also asked if they had binge drunk (defined here as consumed 6 or more alcoholic drinks on one occasion) in the last 30 days.

#### 1.3.5 Oral care habits

Participants reported if and how often they brush and floss their teeth in a typical week. Both measures of the frequency of oral care behaviours were categorised, because they had multimodal distributions and recommended cut-off points were available (American Dental Association, 2016). The measure of the frequency of teeth brushing was broken into the following categories: not every day, at least once a day but less than twice a day, and at least twice a day. The measure of the frequency of flossing was broken into the following categories: never flosses, floss on occasion (1 to 4 times a week), floss on most days (5 to 7 times a week), and floss more than once a day (>7 times a week). The American Dental Association recommends people brush their teeth twice a day and floss once a day to prevent plaque build-up, tooth decay and gum disease (American Dental Association, 2016). The categories *flossing 5 to 7 times a week* and *brushing at least twice a day* most closely matched the recommended guidelines and were selected as the reference category.

### 1.4 Covariates

#### 1.4.1 Childhood socioeconomic status

Childhood SES was a z-transformed composite variable of parental income, education, and occupation status, which was derived by Herrnstein and Murray. Higher scores on the childhood SES variable indicate a more advantaged socio-economic position (Herrnstein & Murray, 1994).

#### 1.4.2 Adult socioeconomic status

Adult SES was a z-transformed composite measure of total net family income and occupation status, as recorded in 2012. To be consistent with Herrnstein and Murray (1994), income was logged and z-transformed. It was also truncated to a minimum value of −4 for income below $3162.45, which is the 2012 income equivalent to $1000 in 1979. Data on occupation were recorded in the US 3-digit, 2000 census codes (Bureau of Labor Statistics, 2015). These codes were then used to derive the occupational status hierarchy by using a scale that was developed by Hauser and Warren (1996). Hauser and Warren (1996) is an updated version of the 1960 Duncan SEI scale that was used by Herrnstein and Murray (1994) (Frederick, 2010). Occupation status received a log transformation and was then z-scored.

### 1.5 Analyses

To test for linearity, generalized linear regression models were used for the continuous outcome measures due to the skewed distribution of their scores. These models were based on either the gamma or Poisson distribution, depending on whichever distribution resulted in a better fitting model. Binary and multinomial logistic regression models were used for the categorised scores.

The associations between IQ and whether or not participants were able to engage in each type of exercise and, for those who could do the activity, the frequency with which each exercise was engaged in were analysed. Another set of analyses analysed the associations between IQ and whether or not each diet-related behaviour was engaged in and, for those who reported they did engage in the behaviour, the frequency with which each dietary behaviour was engaged in. Two additional analyses tested for an association between IQ in youth and how often participants read nutrition information and/or ingredients on food packing. Further analyses tested for a link between IQ and whether or not participants smoke and/or drink and, for those who engage in the respective habit, if they binge drink and the frequency with which they smoke and/or drink. The final set of analyses tested the relationship between IQ and whether or not participants flossed their teeth and, if they did floss, how frequently they flossed. The relationship between IQ and how often participants brush their teeth was also tested.

For each of these analyses, there was a baseline model, which adjusted for age, sex, and ethnicity, a second model that also adjusted for child SES, and a fully adjusted model, which also adjusted for adulthood SES. Childhood and adult SES were adjusted for because studies have found childhood SES to confound some of the IQ-health behaviour associations (Batty, Deary, Schoon, & Gale, 2006; O'Callaghan et al., 2012; Osler et al., 2008; Wennerstad et al., 2010) but other studies did not (Batty, Deary, & Macintyre, 2006; Sjölund, Hemmingsson, Gustafsson, & Allebeck, 2015). Some studies have found adult SES to partially mediate some IQ versus health behaviour associations (Batty et al., 2008; Batty, Deary, Schoon, & Gale, 2006; Clouston et al., 2015; Daly & Egan, 2017; Modig & Bergman, 2012; Sabbah & Sheiham, 2010).

The median of the pooled linear correlations and the estimated effect sizes, calculated from the odds ratios (Chinn, 2000), were used to estimate the pooled summary effect size of the overall association between IQ and health behaviours.

## 2 Results

### 2.1 Characteristics of the sample

The original NLSY-79 sample consisted of 12,686 respondents. The present study is based on the 5347 members who participated in the 2012 survey, 52% were female. The average age of the participants at the time of interview was 51.7 years. Details of the respondents to the 2012 survey, including the percentage with missing data on all our variables of interest, are shown in Table 1. The analyses that follow are based on those with complete data for AFQT, age at interview, sex, ethnicity, childhood SES, adult SES and the relevant health behaviour.

**Table 1:** Demographic characteristics, childhood/adult SES, intelligence scores, and health behaviour information of NLSY-79 study participants in middle age.

		Obs (%)	Missing^a^	Mean	SD
**Demographic information**					
Age		7301	0	51.66	2.25
*Sex*					
Male		3524 (48)	0		
Female		3777 (52)	0		
*Ethnicity*					
Hispanic		1319 (19)	0		
Black		2220 (31)			
Non-Black non-Hispanic		3461 (50)			
**AFQT**		7000	301	−0.33	1.02
**SES**					
Adult SES		5621	1680	0.06	0.81
Income		6107	1194	$78,212	$82,571
Occupation Status		6683	618	35.98	13.74
Childhood SES		7000	301	−0.36	1.08
**Exercise:**					
Are you able to engage in strength training	Yes	6423 (95)	514		
	No	364 (5)			
Are you able to engage in moderate cardio activity	Yes	6865 (98)	266		
	No	170 (2)			
Are you able to engage in vigorous cardio activity	Yes	6669 (95)	300		
	No	332 (5)			

Frequency of strength training (times/week)		6608	994	1.57	2.72
Amount of moderate cardio activity (minutes/week)		6841	782	258.57	515.73
Amount of vigorous cardio activity (minutes/week)		6382	919	177.68	270.15
**Diet**					
Did you have fast food in the past week?	Yes	4266 (59)	15		
	No	3020 (41)			
Did you snack between meals in the past week?	Yes	6123 (84)	42		
	No	1136 (16)			
Did you skip any meals in the past week?	Yes	4433 (61)	37		
	No	2831 (39)			
Did you have any sugary drinks in the past week?	Yes	3862 (53)	20		
	No	3419 (47)			

Number of times you eat fast food (past week)		7286	15	1.26	1.72
Number of times you snack between meals (past week)		7259	42	6.20	6.41
Number of meals you skip (past week)		7263	37	2.21	2.68
Number of sugary drinks (past week)		7281	20	3.22	6.34

**When shopping do you:**					
Read nutritional information	Always	2144	60		
	Often	1509			
	Sometimes	1543			
	Rarely	719			
	Never	1326			

Read the ingredients	Always	1873	59		
	Often	1278			
	Sometimes	1737			
	Rarely	861			
	Never	1493			
**Drinking & Smoking**					
Did you drink any alcohol in the past month?	Yes	3978 (55)	39		
	No	3284 (45)			
Did you have 6 or more drinks on one occasion in the past month?	Yes	1034 (26)	3330		
	No	2937(74)			
Do you smoke cigarettes?	Yes	1815 (25)	49		
	No	5437 (75)			
Number of alcoholic drinks (per week)		7242	59	2.98	6.85
Number of cigarettes (per day)		1367	18	13.77	8.00
**Oral Health Care**					
Do you brush your teeth?	Yes	6861 (99.75)	423		
	No	17 (0.25)			
Do you floss?	Yes	5289 (77)	435		
	No	1577 (23)			
Brush teeth (times per week)		6855	446	13.66	5.18
Floss (times per week)		6858	443	5.41	5.67

### 2.2 Intelligence in relation to health behaviours

Table 2 shows the distribution of the survey respondents across each health behaviour outcome category and by AFQT tertile. In each case, there is a significant difference in the distribution across IQ tertile by outcome category. In many cases, those who reported engaging in less healthy behaviours tended to be in a lower IQ tertile and vice-versa. Supplementary table S1 shows how intelligence and childhood/adult SES characteristics vary across the outcome categories for each of the health behaviours. In most cases, higher intelligence and higher childhood/adult SES were associated with healthier behaviours.

**Table 2:** Distribution of the survey respondents across each health behaviour outcome categories by AFQT tertile.

		AFQT Tertile [obs (%)]			Chi square (*p* value)
		1	2	3	
Mean (SD) AFQT score		−1.36 (0.49)	−0.28 (0.26)	0.87 (0.55)	
**Are you able to do the following activities?**					
Strength training activities	Yes	2218 (35.9)	1988 (32.3)	1957 (31.8)	60.4 (*p* < .001)
	No	187 (54.7)	111 (31.9)	50 (14.4)	

Moderate cardiovascular activities	Yes	2372 (36.0)	2143 (32.6)	2067 (31.4)	45.98 (*p* < .001)
	No	99 (60.7)	43 (26.4)	21 (12.9)	

Vigorous cardiovascular activities	Yes	2293 (35.8)	2075 (32.4)	2030 (31.7)	64.6 (p < .001)
	No	176 (55.7)	97 (30.7)	43 (13.6)	
**How much of the following activities do you do?**					
Strength training (sessions/week)	0	1312 (39.1)	1109 (32.9)	942 (28.0)	102.2 (*p* < .001)
	1 to 3	476 (27.8)	537 (31.4)	698 (40.8)	
	≥4	377 (38.4)	305 (31.1)	299 (30.5)	

Moderate cardio vascular activity (minutes/week)	<75	1051 (37.7)	883 (31.7)	853 (30.6)	66.7 (p < .001)
	75 to 149	343 (30.2)	362 (31.9)	431 (37.9)	
	150 to 509	538 (34.1)	500 (31.7)	542 (34.3)	
	510	306 (40.9)	279 (37.3)	164 (21.9)	

Vigorous cardiovascular activity (minutes/week)	<38	886 (40.9)	665 (30.7)	613 (28.3)	119.7 (p < .001)
	38 to 74	201 (28.8)	239 (34.2)	258 (36.9)	
	75 to 254	562 (27.9)	671 (33.4)	776 (38.6)	
	255	498 (39.6)	420 (33.4)	340 (27.0)	
**In the past week have you:**					
Eaten fast food	Yes	1542 (37.7)	1378 (33.7)	1175 (28.7)	19.81 (p < .001)
	No	1052 (36.4)	872 (30.2)	968 (33.5)	

Skipped any meals	Yes	1485 (34.9)	1439 (33.8)	1333 (31.3)	25.90 (p < .001)
	No	1104 (40.8)	803 (29.7)	801 (29.6)	

Snacked between meals	Yes	2098 (35.6)	1884 (32.0)	1906 (32.4)	58.64 (p < .001)
	No	484 (45.1)	360 (33.6)	229 (21.3)	

Had any sugary drinks	Yes	1617 (43.8)	1210 (32.8)	868 (23.5)	227.41 (p < .001)
	No	973 (29.6)	1038 (31.6)	1275 (38.8)	
**When shopping do you:**					
Read nutritional information	Always	642 (31.2)	698 (33.9)	721 (34.9)	417.4 (p < .001)
	Often	352 (24.0)	448 (30.6)	665 (45.4)	
	Sometimes	666 (45.3)	463 (31.5)	342 (23.3)	
	Rarely	258 (37.2)	239 (34.5)	196 (28.3)	
	Never	656 (52.2)	388 (30.9)	212 (16.9)	

Read the ingredients	Always	643 (35.7)	593 (32.9)	565 (31.4)	234.8 (p < .001)
	Often	330 (26.7)	387 (31.3)	521 (42.1)	
	Sometimes	650 (39.1)	504 (30.3)	509 (30.6)	
	Rarely	259 (31.2)	284 (34.3)	286 (34.5)	
	Never	693 (48.9)	469 (33.1)	255 (18.0)	
**Smoking & Drinking**					
Did you drink alcohol in the past month?	Yes	1066 (27.8)	1277 (33.3)	1487 (38.8)	373.96 (p < .001)
	No	1506 (48.1)	970 (30.9)	655 (20.9)	

Did you have 6 or more drinks on one occasion in the past month?	Yes	357 (36.0)	321 (32.4)	313 (31.6)	50.8 (p < .001)
	No	706 (24.9)	956 (33.8)	1170 (41.3)	

Do you smoke?	Yes	835 (47.8)	580 (33.2)	331 (18.9)	176.9 (p < .001)
	No	1752 (33.6)	1661 (31.8)	1807 (34.6)	
**Oral care**					
Do you floss?	Yes	1640 (32.3)	1693 (33.3)	1752 (34.5)	116.9 (p < .001)
	No	705 (46.8)	444 (29.5)	356 (23.7)	

How often do you floss (times per week)?	Never	705 (46.8)	444 (29.5)	357 (23.7)	177.2 (p < .001)
	1 to 4	541 (29.6)	606 (33.2)	678 (37.2)	
	5 to 7	668 (30.9)	694 (32.1)	798 (36.9)	
	>7	431 (39.2)	393 (35.7)	276 (25.1)	

How many times a day do you brush your teeth?	< once	109 (57.9)	44 (23.4)	35 (18.6)	69.5 (p < .001)
	1 to <2	633 (40.3)	479 (30.5)	458 (29.2)	
	2+	1608 (33.2)	1620 (32.5)	1617 (33.4)	

**Fig. 1:**
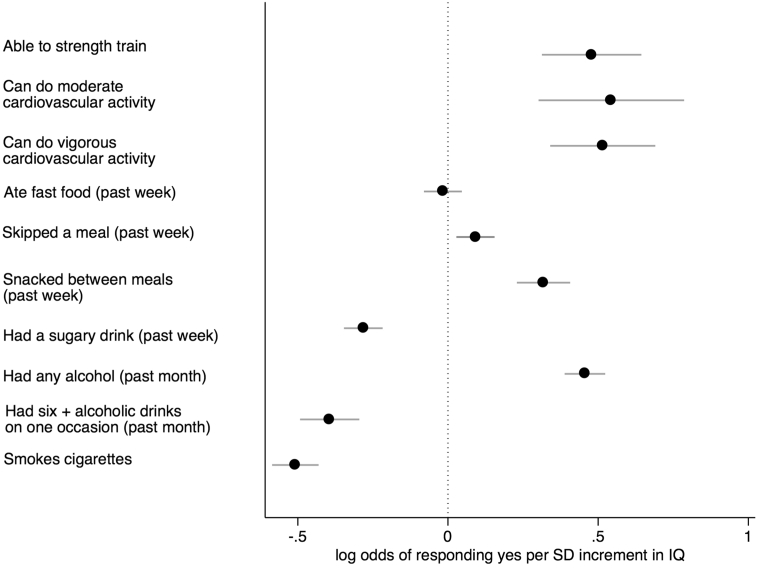
Log odds of responding yes to each health behaviour question, in middle age, per SD increment in intelligence in youth, adjusted for age at the time of interview, sex, and ethnicity.

**Fig. 2:**
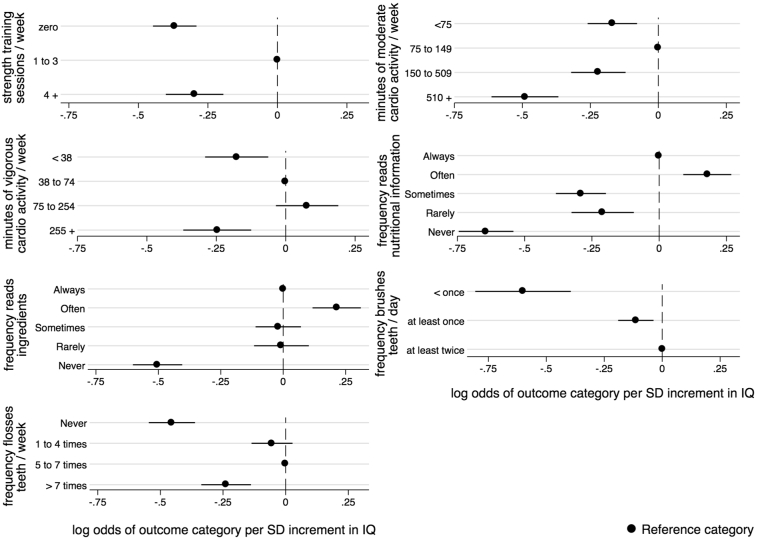
Log odds of each outcome category for different types of exercise habits, nutritional literacy habits, and oral hygiene habits, in middle age, per SD increment in IQ in youth, adjusted for age at the time of interview, sex, and ethnicity.

Table 3 shows the ORs for the relationship between intelligence in youth and physical activity in middle age, both with and without adjustment for childhood SES and adult SES. The results of the likelihood ratio tests confirm the overall statistical significance of the association between IQ and the three measures of the frequency of activity. The results of the logistic regressions found that higher intelligence is associated with increased odds of being able to engage in all three kinds of physical activity. After adjusting for age, sex, and ethnicity in the baseline model, the odds ratio for being able to engage in the activity ranged from 1.61 (95% CI 1.37 to 1.90) for strength training to 1.72 (95% CI 1.35 to 2.20) for moderate cardiovascular activity (Fig. 1). Adjusting for childhood SES resulted in little change in the estimated effect sizes. Adjusting for adult SES reduced the size of the ORs to 1.40 and 1.46, respectively, but all three associations remained significant. The results in Table 3 also show that, across all three types of exercise, a SD increment in IQ was associated with being less likely to be inactive and with being less likely to do a lot of activity (Fig. 2). Relative to doing a medium amount of strength training each week, a SD increment in IQ was associated with being less likely to not do any (OR 0.69, 95% CI 0.64 to 0.75) and with being less likely to do four or more sessions (OR 0.74, 95% CI 0.67 to 0.82) per week, in the baseline model. Adjusting for childhood SES led to slight attenuation of the effect sizes. Upon adjusting for adult SES, the size of the ORs were reduced but the majority of associations remained significant. The extent of the attenuation was heterogeneous across outcome category for amount of strength training and vigorous activity. For example, compared to doing a medium amount of strength training there was more attenuation of the estimated effect size for doing no sessions per week (71%) than there was for doing four or more sessions per week (12%).

**Table 3:** Logistic and multinomial logistic regression analyses of the relation between an SD increment in IQ in youth and strength training, moderate cardiovascular activity and vigorous cardiovascular activity in middle age, with adjustment for potential confounding and mediating variables.

Exercise habits			n	Model 1		LR chi-square	Model 2	LR chi-square		Model 3		LR chi-square
				OR (95% CI)	P value	(p value)	OR (95% CI)	(p value)	(p value)	OR (95% CI)	P value	(p value)
**Are you able to engage in the following activities?**												
Strength training		Yes	4776	1.61 (1.37 to 1.90)	p < .001		1.61 (1.34 to 1.93)	*p* < .001		1.40 (1.14 to 1.71)	p = .001	
		No	213									

Moderate cardiovascular activity		Yes	5091	1.72 (1.35 to 2.20)	p < .001		1.71 (1.32 to 2.25)	p < .001		1.46 (1.09 to 1.96)	*p* = .011	
		No	97									

Vigorous cardiovascular activity		Yes	4963	1.67 (1.41 to 1.99)	p < .001		1.71 (1.40 to 2.07)	p < .001		1.33 (1.08 to 1.65)	p < .001	
		No	188									
**In a typical week, how much of the following activities do you do?**												
Strength training (sessions/week)	inactive	0	2552	0.69 (0.64 to 0.75)	p < .001	158.67	0.74 (0.68 to 0.81)	*p* < .001	179.90	0.91 (0.82 to 0.99)	*p* = .045	286.14
	medium	1 to 3	1381	ref		(p < .001)	ref		(p < .001)	ref		(p < .001)
	a lot	4	763	0.74 (0.67 to 0.82)	p < .001		0.73 (0.66 to 0.83)	p < .001		0.77 (0.68 to 0.88)	p < .001	

Moderate cardiovascular activity (minutes/week)	inactive	<75	2151	0.84 (0.77 to 0.92)	p < .001	144.54	0.86 (0.78 to 0.96)	p < .001	146.42	0.87 (0.78 to 0.97)	*p* = .014	203.47
	a little	75 to 149	891	ref		(p < .001)	ref		(p < .001)	ref		(p < .001)
	medium	150 to 509	1226	0.80 (0.73 to 0.89)	p < .001		0.80 (0.72 to 0.90)	p < .001		0.86 (0.76 to 0.97)	p = .014	
	a lot	510	574	0.61 (0.54 to 0.69)	p < .001		0.63 (0.54 to 0.72)	p < .001		0.76 (0.65 to 0.88)	p < .001	

Vigorous cardiovascular activity (minutes/week)	inactive	<38	1601	0.84 (0.75 to 0.94)	*p* = .002	230.80	0.85 (0.75 to 0.96)	p = .011	247.12	0.95 (0.83 to 1.09)	*p* = .453	320.56
	a little	38 to 74	560	ref		(p < .001)	ref		(*p* < .001)	ref		(p < .001)
	medium	75 to 254	1647	1.08 (0.97 to 1.21)	*p* = .179		1.03 (0.90 to 1.16)	*p* = .680		0.96 (0.84 to 1.10)	*p* = .571	
	a lot	255	964	0.78 (0.69 to 0.88)	p < .001		0.73 (0.63 to 0.84)	*p* < .001		0.73 (0.63 to 0.85)	p < .001	

Note. Model 1 = adjustment for age at interview, sex, and ethnicity; Model 2 = model 1 + Childhood SES; Model 3 = + Adult SES.

**Fig. 3:**
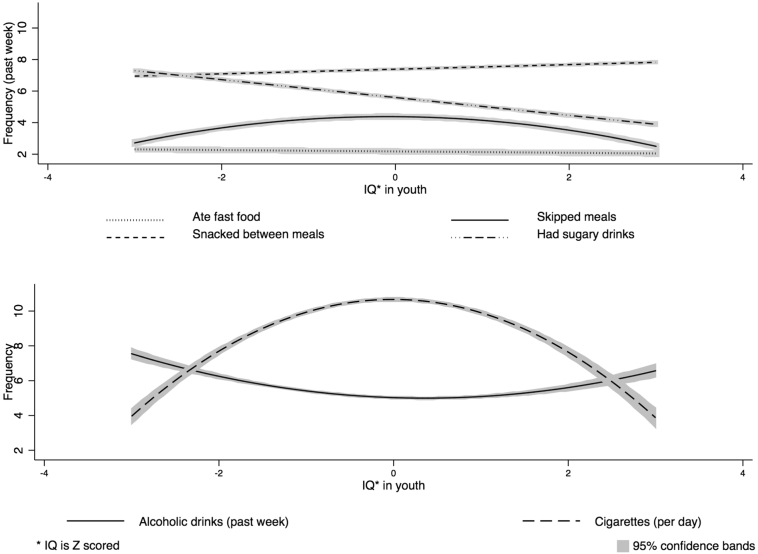
Fitted estimates of the association between intelligence in youth and diet related behaviours in the past week, the number of cigarettes smoked (per day) and alcoholic drinks consumed (per week) in middle age, adjusted for age at the time of interview, sex, and ethnicity.

The results of the logistic and generalized linear regression analyses between intelligence and dietary habits are displayed in Table 4. In the baseline model, higher IQ scores were associated with being more likely to skip a meal (OR 1.10, 95% CI 1.03 to 1.17) and snack between meals (OR 1.37, 95% CI 1.26 to 1.50), and less likely to have had a sugary drink (OR 0.75, 95% CI 0.71 to 0.80) in the past week (Fig. 1). IQ was not associated with the frequency of eating fast food or snacking between meals over the past week. In the basic model, a SD increment in IQ was associated with drinking fewer sugary drinks in the past week (B = −0.15, 95% CI -0.20 to −0.10) (adjusting for age, sex, and ethnicity) (Fig. 3). A non-linear association was found between IQ in youth and the number of meals that were skipped in the past week in the baseline model. Higher IQ was associated with skipping more meals for the lower half of the IQ range but it was associated with skipping fewer meals for the upper half of IQ scores (Fig. 3). Adjusting for childhood SES resulted in little change in any of the effect sizes. Adjusting for adult SES attenuated the effect for having had a sugary drink and the amount of sugary drinks consumed to the null. The non-linear association was attenuated upon adjusting for adult SES but statistical significance was maintained.

**Table 4:** Logistic and generalized^a^ linear regression analyses of the relation between an SD increment in IQ in youth and four separate dietary habits in middle age, with adjustment for potential confounding and mediating variables.

Dietary habits	n	Model 1		Model 2		Model 3	
		OR (95% CI)	P value	OR (95% CI)	P value	OR (95% CI)	P value
In the past week have you:							
Eaten fast food	Yes (*n* = 3179)	0.98 (0.92 to 1.05)	*p* = .602	1.05 (0.98 to 1.13)	*p* = .180	1.02 (0.94 to 1.10)	*p* = .655
	No (*n* = 2166)						

Skipped any meals	Yes (*n* = 3275)	1.10 (1.03 to 1.17)	p = .005	1.10 (1.02 to 1.18)	p = .011	1.16 (1.07 to 1.26)	p < .001
	No (*n* = 2059)						

Snacked between meals	Yes (*n* = 4557)	1.37 (1.26 to 1.50)	p < .001	1.47 (1.33 to 1.62)	p < .001	1.38 (1.24 o 1.55)	p < .001
	No (*n* = 780)						

Had any sugary drinks	Yes (*n* = 2786)	0.75 (0.71 to 0.80)	p < .001	0.85 (0.79 to 0.91)	p < .001	0.96 (0.89 to 1.03)	*p* = .304
	No (*n* = 2555)						

		**B (95% CI)**	**P value**	**B (95% CI)**	**P value**	**B (95% CI)**	**P value**
In the past week how many times have you^a^, ^b^:							
Eaten fast food	3179	(−0.02 (−0.06 to 0.01)	*p* = .171	−0.03 (−0.06 to 0.01)	*p* = .165	(−0.02 (−0.06 to 0.02)	*p* = .409

Skipped meals	3275	(−0.04 (−0.07 to 0.1)	*p* = .004	−0.05 (−0.08 to −0.01)	p = .004	(−0.02 (−0.05 to 0.01)	*p* = .270
		*−0.05 (−0.06 to* *−* *0.03)*^c^	*p* *<* *.001*	*−0.05 (−0.06 to* *−* *0.03)*^*c*^	*p* *<* *.001*	*−0.05 (−0.06 to* *−* *0.03)*^*c*^	*p* *<* *.001*

Snacked between meals	4557	0.003 (−0.03 to 0.03)	*p* = .827	−0.001 (−0.03 to 0.03)	*p* = .935	0.02 (−0.02 to 0.05)	*p* = .285

Had sugary drinks	2786	(−0.15 (−0.20 to −0.10)	*p* < .001	−0.13 (−0.19 to −0.07)	*p* < .001	(−0.03 (−0.10 to 0.04)	*p* = .392

Note. Model 1 = adjustment for age at interview, sex, and ethnicity; Model 2 = model 1+ Childhood SES; Model 3 = + Adult SES.

a All generalized linear models based on gamma distribution and log link.

b Analyses are based on those who said they had engaged in the respective behaviour.

c Italicized results are for the quadratic terms.

Table 5 shows the ORs from the multinomial logistic regression between intelligence and reading food labels. In the basic model (adjusting for age, sex, and ethnicity), higher IQ scores were associated with being more likely to read both nutritional information (OR 1.20, 95% CI 1.09 to 1.31) and ingredients (OR 1.24, 1.12 to 1.36) often, compared to always, and with being less likely to never read both nutritional information (OR 0.53, 95% CI 0.47 to 0.58) and ingredients (OR 0.60, 95% 0.55 to 0.67) (Fig. 2). Adjusting for childhood SES resulted in little change in effect sizes. Adjusting for adult SES led to slightly more attenuation, with the majority of associations remaining significant. The results of the likelihood ratio tests confirm the overall statistical significance of the association between IQ and the frequency of reading food labels.

**Table 5:** Multinomial logistic regression analysis of a SD increment in IQ in youth and two indicators of nutritional literacy in middle age, with adjustment for potential confounding and mediating factors.

Nutritional literacy habits		n	Model 1	p value	LR chi-square	Model 2		LR chi-square	Model 3		LR chi-square
			OR (95% CI)		(p value)	OR (95% CI)	p value	(p value)	OR (95% CI)	p value	(p value)
Do you read nutritional information when shopping for a new item?	Always	1585	ref		643.31	ref		657.05	ref		709.27
	Often	1181	1.20 (1.09 to 1.31)	p < .001	(p < .001)	1.23 (1.11 to 1.36)	p < .001	(p < .001)	1.24 (1.11 to 1.38)	p < .001	(p < .001)
	Sometimes	1101	0.75 (0.68 to 0.82)	p < .001		0.80 (0.73 to 0.90)	p < .001		0.88 (0.78 to 0.98)	*p* = .023	
	Rarely	539	0.81 (0.72 to 0.91)	p < .001		0.86 (0.76 to 0.98)	*p* = .020		0.95 (0.83 to 1.09)	*p* = .515	
	Never	911	0.53 (0.47 to 0.58)	p < .001		0.57 (0.51 to 0.63)	p < .001		0.66 (0.59 to 0.75)	p < .001	

Do you read the ingredients when shopping for a new item?	Always	1343	ref		450.94	ref		468.14	ref		488.89
	Often	1001	1.24 (1.12 to 1.36)	p < .001	(p < .001)	1.31 (1.17 to 1.46)	p < .001	(p < .001)	1.25 (1.11 to 1.41)	p < .001	(p < .001)
	Sometimes	1272	0.98 (0.90 to 1.07)	*p* = .670		1.07 (0.96 to 1.19)	*p* = .212		1.08 (0.97 to 1.21)	*p* = .148	
	Rarely	655	0.99 (0.89 to 1.11)	*p* = .900		1.08 (0.96 to 1.23)	*p* = .202		1.06 (0.93 to 1.22)	*p* = .368	
	Never	1047	0.60 (0.55 to 0.67)	p < .001		0.65 (0.58 to 0.72)	p < .001		0.70 (0.62 to 0.79)	p < .001	

Note. Model 1 = adjustment for age at interview, sex, and ethnicity; Model 2 = model 1 + Childhood SES; Model 3 = + Adult SES.

The ORs and Bs from the analyses between intelligence and drinking and smoking behaviours are presented in Table 6. In the baseline model (adjusting for age, sex, and ethnicity), a SD increment in intelligence in youth was associated with increased odds of having had a drink (OR 1.58, 95% CI 1.47 to 1.69) in the past month and with reduced odds of having had >6 drinks on one occasion (OR 0.67, 95% CI 0.61 to 0.74) in the past 30 days (Fig. 1). Adjusting for adult SES resulted in marked attenuation of both estimated effect sizes, though statistical significance at conventional levels was maintained in both cases. A non-linear association was found between a SD increment in IQ and number of drinks consumed per week, excluding those who don't drink, in the baseline model (adjusted for age, sex, and ethnicity) (Fig. 3). Higher IQ was associated with having fewer drinks per week for the lower half of IQ scores. For the upper half of IQ scores, higher IQ was associated with having more alcoholic drinks per week. Including childhood SES in the model resulted in no attenuation, and including adult SES only slightly reduced the effect sizes.

**Table 6:** Logistic and generalized linear regression of a SD increment in IQ in youth and drinking and smoking behaviour in middle age, with adjustment for potential mediating and confounding variables.

Smoking and Drinking habits	Model 1			Model 2		Model 3	
	obs	OR (95% CI)	P value	OR (95% CI)	P value	OR (95% CI)	P value
**Do you smoke/drink/binge drink?**							
Have you drunk alcohol in the last 30 days?	Yes (*n* = 3115)	1.58 (1.47 to 1.69)	p < .001	1.39 (1.29 to 1.50)	p < .001	1.23 (1.14 to 1.34)	p < .001
	No (*n* = 2221)						

Have you had 6 or more drinks in one occasion in the past 30 days?	Yes (*n* = 801)	0.67 (0.61 to 0.74)	p < .001	0.71 (0.63 to 0.79)	p < .001	0.83 (0.74 to 0.94)	*p* = .003
	No (*n* = 2311)						

Do you smoke?	Yes (*n* = 1268)	0.60 (0.56 to 0.65)	p < .001	0.61 (0.56 to 0.66)	p < .001	0.81 (0.74 to 0.90)	p < .001
	No (*n* = 4058)						

	B (95% CI)		B (95% CI)		B (95% CI)		
**How much do you smoke/drink?**							
How many alcoholic drinks do you have in a typical week?	3109	(−0.11 (−0.13 to −0.10)	p < .001	−0.12 (−0.14 to −0.10)	*p* < .001	(−0.04 (−0.07 to −0.02)	p < .001
		*0.02 (0.01 to 0.04)*^a^	*p* *<* *.001*	*0.03 (0.01 to 0.04)*^a^	*p* *<* *.001*	*0.02 (0.01 to 0.03)*^a^	*p* *<* *.001*

How many cigarettes do you smoke per day?	1259	(−0.15 (−0.17 to −0.12)	p < .001	−0.13 (−0.16 to −0.10)	p < .001	(−0.11 (−0.15 to −0.08)	p < .001
		*−0.08 (−0.10 to* *−* *0.06)*^a^	*p* *<* *.001*	*−0.08 (−0.09 to* *−* *0.06)*^*a*^	*p* *<* *.001*	*−0.08 (−0.09 to* *−* *0.06)*^*a*^	*p* *<* *.001*

Note. Model 1 = adjustment for age at interview, sex, and ethnicity; Model 2 = model 1 + Childhood SES; Model 3 = + Adult SES.

Generalized linear models based on Poisson distribution and log link.

a Italicized results are for the quadratic terms.

In the baseline model, a SD increment in intelligence in youth was associated with reduced odds of being a smoker (OR 0.60, 95% CI 0.56 to 0.65) (Table 6 & Fig. 1). A non-linear association was found between a SD increment in IQ and number of cigarettes smoked a day, excluding those who do not smoke (Table 6 & Fig. 3). A higher IQ is associated with smoking more cigarettes a day for the lower half of IQ scores and it is associated with smoking fewer cigarettes a day for the upper half of IQ scores. Adjusting for childhood SES had almost no impact on the estimated effect sizes. Adjusting for adult SES resulted in slight attenuation but statistical significance was maintained.

Table 7 shows the results of the analyses between intelligence and oral health care behaviours. Adjusting for age, sex, and ethnicity, a SD increment in IQ was associated with being more likely to floss (OR 1.47, 95% CI 1.35 to 1.59). In the baseline model, compared to flossing 5 to 7 times a week, a SD increment in IQ was associated with being less likely to never floss (OR 0.64, 95% CI 0.58 to 0.70) and to floss more than once a day (OR 0.79, 95% CI 0.71 to 0.87) (Fig. 2). In the baseline model (adjusting for age, sex, and ethnicity), higher IQ was associated with reduced odds of brushing less than twice a day (OR 0.90, 95% CI 0.83 to 0.96) and less than once a day (OR 0.55, 95% 0.44 to 0.68), compared to brushing teeth at least twice a day (Fig. 2). Including childhood SES led to some attenuation, most notably for the frequency of brushing teeth. Including adult SES in the model led to attenuation, and the associations between IQ and frequency of teeth brushing were no longer significant.

**Table 7:** Logistic and generalized linear regression analysis of a SD increment in IQ in youth and preventative oral health care behaviours in middle age, with adjustment for potential mediating and confounding variables.

Oral care habits		Model 1		LR chi square	Model 2		LR chi square	Model 3		LR chi Square
		OR (95% CI)	p value	(p value)	OR (95% CI)	p value	(p value)	OR (95% CI)	p value	(p value)
Do you floss?	Yes (*n* = 4010)	1.47 (1.35 to 1.59)	p < .001		1.41 (1.29 to 1.54)	p < .001		1.18 (1.07 to 1.30)	p = .001	
	No (*n* = 1083)									

How often do you floss?	Never (n = 1083)	0.64 (0.58 to 0.70)	p < .001	337.54	0.68 (0.61 to 0.75)	p < .001	346.34	0.83 (0.74 to 0.93)	p = .001	433.24
	1 to 4 times a week (*n* = 1468)	0.95 (0.87 to 1.03)	*p* = .193	(p < .001)	0.99 (0.90 to 1.08)	*p* = .843	(p < .001)	1.05 (0.95 to 1.16)	*p* = .361	(p < .001)
	5 to 7 times a week (*n* = 1705)	ref			ref			ref		
	More than once a day (*n* = 837)	0.79 (0.71 to 0.87)	p < .001		0.80 (0.72 to 0.90)	p < .001		0.84 (0.74 to 0.94)	P = .003	

How many times a day do you brush your teeth^a^	Less than once (*n* = 125)	0.55 (0.44 to 0.68)	p < .001	199.78	0.65 (0.52 to 0.82)	p < .001	217.57	0.84 (0.65 to 1.07)	*p* = .155	268.32
	At least once but less than twice (*n* = 1198)	0.90 (0.83 to 0.96)	p = .003	(p < .001)	0.94 (0.87 to 1.03)	*p* = .192	(p < .001)	1.06 (0.96 to 1.16)	*p* = .252	(p < .001)
	at least twice (*n* = 3778)	ref			ref			ref		

Note. Model 1 = adjustment for age at interview, sex, and ethnicity; Model 2 = model 1 + Childhood SES; Model 3 = + Adult SES.

a Only 17 participants reported that they did not brush their teeth so we did not analyse the association between AFQT and whether or not people brushed their teeth.

The median of the pooled linear correlations and the estimated effect sizes, calculated from the odds ratios (Chinn, 2000) was used as a measure of the pooled summary estimate of the overall association between IQ and health behaviours. This pooled estimate was 0.12 with a standard error of 0.03.

## 3 Discussion

In the present study, higher intelligence in youth was associated with a number of different healthy behaviours in middle age. Looking first at physical activity, higher intelligence was associated with both being more likely to be able to engage in all three kinds of physical activity and with being less likely to take either none or a lot of each type of activity, relative to taking some activity. Looking at the results for oral health habits, higher IQ was linked with being more likely to brush at least twice a day and to floss on most days. Higher IQ was also linked with reading the ingredients and nutritional information when shopping for new food items often compared to always. Higher intelligence was also linked with being less likely to smoke, less likely to consume 6 or more alcoholic drinks on one occasion, and with consuming fewer sugary drinks.

A number of novel associations were also found between higher intelligence and behaviours that may or may not be linked with poorer health outcomes (i.e. skipping meals and snacking between meals). Higher intelligence was linked with being more likely to have skipped a meal and snacked between meals. An inverted-u shaped association was also found between higher intelligence and the number of meals skipped. Higher IQ was linked with skipping more meals for the lower half of the IQ scores but it was associated with skipping fewer meals for the upper half of the IQ range. The health consequences of skipping meals and snacking between meals are not well understood. Mesas, Muñoz-Pareja, López-García, and Rodríguez-Artalejo (2012) conducted a systematic review of studies that investigated the association between selected eating behaviours (including skipping meals and snacking between meals) and BMI. They concluded that there is inconsistent evidence of the relationship between these eating behaviours and BMI. For instance, out of the 15 studies that tested the association between skipping meals, mainly breakfast, and BMI in adulthood, 10 studies found it was linked with a higher BMI but five studies found no association. The only eating behaviour that had a consistent relationship with BMI was snacking. The nine studies that investigated this association found snacking was linked with a higher BMI. Mesas et al. (2012) did not run a meta-analysis due to the heterogeneity of the measures and models that were included in the reviewed studies, including those studies that looked at skipping meals and snacking between meals. The inverted U-shaped association between IQ and number of meals skipped is difficult to interpret because only those who reported they had skipped a meal were asked about number of meals skipped so some respondents may have been differentially excluded from the analytical sample.

Associations were also found between IQ and behaviours that are known to be linked with poor health outcomes (i.e. drinking alcohol and the number of cigarettes smoked).

First, higher intelligence was linked with being more likely to have consumed alcohol in the past 30 days. A u-shaped association was found between intelligence and the number of alcoholic drinks consumed a week. The results suggest a different pattern of drinking for those with a higher versus lower intelligence. The former may engage in more moderate drinking behaviours and drink frequently throughout the week but only have few units on each occasion. The latter may engage in more risky drinking behaviours and drink infrequently throughout the week but consume a high amount of alcohol on each occasion.

The J-shaped relationship that is observed between alcohol consumption and premature mortality was thought to suggest that low to moderate levels of alcohol consumption had health protective benefits (O'Keefe et al., 2012), however this is no longer thought to be the case. More recently, the results from a mendelian randomisation meta-analysis of 56 epidemiological studies (*n* = 261,991) suggests that abstaining from drinking alcohol and, amongst drinkers, lower levels of alcohol consumption is linked with a reduced risk of cardiovascular disease. There was no evidence to support the J-shaped link between alcohol consumption and mortality (Holmes et al., 2014). Alcohol consumption is also linked with cancer. Pelucchi, Tramacere, Boffetta, Negri, and Vecchia (2011) reviewed the literature on the links between alcohol consumption and different cancers. They found that both high and low weekly alcohol consumption increases the risk of cancer, compared to abstainers or occasional drinkers. For example, high amounts of alcohol consumption (>4 drinks a day) is linked with an increased risk of oral and pharynx cancer (relative risk (rr, 95% CI) 5.24, 95% CI 4.36 to 6.30), based on 31 studies, and cancer of the oesophagus (4.89, 3.84 to 6.23), based on 39 studies. Low amounts of alcohol also increased the risk of these cancers with a relative risk of 1.21 (95% CI 1.10 to 1.33), based on 20 studies, and 1.31 (95% CI 1.10 to 1.57), based on 26 studies, respectively. The evidence suggests that both high and low amounts of alcohol consumption can increase the risk of cardiovascular disease and cancer.

Second, the inverted-u shaped association between intelligence and the number of cigarettes smoked suggests that, for those in the lower 50% of IQ scores, a SD increment in IQ is linked with smoking more cigarettes a day and for those in the higher 50% of IQ scores, a SD increment in IQ is linked with smoking fewer cigarettes a day. Cigarette smoking is linked with multiple adverse health risk (Saha, Bhalla, Whayne, & Gairola, 2007). Those in the upper 50% of IQ scores may believe that smoking fewer cigarettes is less detrimental to health. A recent systematic review with a meta analysis of 141 studies found that smoking just one cigarette a day, relative to smoking 20 a day, carries with it a notable increased risk of both coronary heart disease and stroke. For example, men and women who smoked just one cigarette a day had 46% and 31% of the excess risk, respectively, of developing coronary heart disease of those who smoke 20 cigarettes a day. The article concludes that there are no safe levels of smoking for cardiovascular outcomes (Hackshaw, Morris, Boniface, Tang, & Milkenkovic, 2018). The inverted U-shaped association between IQ and number of cigarettes smoked is difficult to interpret because this association is based only on those who reported they do smoke so some respondents may have been differentially excluded from the analytical sample.

### 3.1 Confounding and mediating

Regarding possible confounding effects of early life circumstances, the associations in the present study appeared to be largely independent of early life socio-economic position. In most cases, adult SES appeared to account for some of the association between IQ and the different health behaviours. There were three notable findings regarding mediation by adult SES. First, after adjusting for adult SES the associations with brushing teeth and sugary drinks were substantially attenuated and were no longer significant. This might suggest that IQ in youth influences these outcomes via adult SES. Second, adjusting for adult SES had no attenuating effect on whether or not people skipped meals or snacked between meals; instead, the effect size slightly increased upon adjustment. This suggests that early life IQ influences these outcomes independently of adult SES. Third, after including adult SES in the models that analysed the amount of strength training and vigorous activity, the extent of the attenuation was different across outcome category. The attenuation was greater for being inactive than it was for doing a lot of activity. For example, the respective attenuations for strength training were 71% compared to 12%.

### 3.2 Comparison with other studies

Many of the present findings are consistent with previous studies that also found higher IQ to be linked with more health enhancing physical activity habits (Batty, Deary, Schoon, & Gale, 2006; Clouston et al., 2015; Osler et al., 2008). The present study is the first that we are aware of to find that higher intelligence in youth was associated with lower chances of being either inactive or doing a lot of activity, compared to taking some activity in middle age. The study by O'Callaghan et al. (2012) found an inverted u-shaped relationship between IQ and amount of vigorous activity, with higher IQ associated with moderate amounts of activity. However, the direction of the relationship could not be determined from their study because both IQ and physical activity was measured at the same time (O'Callaghan et al., 2012). The findings of an association between higher intelligence in youth and moderate amounts of strength training in middle age were novel to the present study.

Of the few previous studies that investigated the association between early life IQ and dietary choices, most found higher IQ to be linked with healthier dietary choices, which is consistent with the present finding that higher IQ was linked with consuming fewer sugary drinks (Batty, Deary, Schoon, & Gale, 2006; Crichton et al., 2015; Kanazawa, 2013; Osler et al., 2008). A study by Lyerly and Reeve (2014) was the most comparable to the present study because it was also based on data from the NLSY-79. The measure of general intelligence used in their study was derived from the first un-rotated principal component of the Armed Services Vocational Aptitude Battery. They included three of the same measures of dietary habits that were in the present study (frequency with which participants consumed fast food and sugary drinks and how often they skipped meals in the past 7 days). Although Lyerly and Reeve reported that intelligence was not associated with any of the dietary habits, the validity of their conclusions are questionable due to the methodological limitations of their study design, which were previously outlined. Consistent with the Lyerly and Reeves study, the present study did not find a link between IQ and eating fast food. Unlike the Lyerly and Reeves study, the present study found a negative association with the frequency of consuming sugary drinks and an inverted-u shaped association with the number of meals skipped. The current findings that those who had higher mental ability in adolescence were more likely to often and less likely to never read food labels when purchasing an item for the first time, compared to always reading this information, were novel to the present study.

The observed associations between higher IQ and being less likely to abstain from drinking (Osler et al., 2008 and less likely to engage in binge drinking (Sjölund, Hemmingsson, Gustafsson, & Allebeck, 2015; Cheng et al., 2010) are consistent with the results observed in previous studies. To the best of our knowledge, the observed U-shaped association between IQ and the amount of alcoholic drinks consumed per week is novel. This non-linear association could help to explain the mixed results observed between IQ and drinking behaviour in previous studies (Sjölund, Hemmingsson, & Allebeck, 2015; Osler et al., 2008; Batty, Deary, & Macintyre, 2006; Clouston et al., 2015; Cheng & Furnham, 2013. The positive association between IQ and alcohol consumption in the upper 50% of IQ scores is consistent with findings of a positive association between IQ and drinking more frequently and having a higher intake of alcohol (Batty, Deary, & Macintyre, 2006; Cheng & Furnham, 2013; Clouston et al., 2015; Osler et al., 2008). The negative association between IQ and alcohol in the lower 50% of scores is consistent with findings lower IQ to be linked with heavy versus light drinking (Sjölund, Hemmingsson, & Allebeck, 2015). A limitation of these previous studies is that they did not look at the number of drinks consumed as a continuous measure, as was done in the present study. Future studies could look for a non-linear IQ-drinking association in cohorts from different countries. There are two primary reasons to investigate this. First, country to country variation in the IQ-drinking behaviour association has been found (Batty, Deary, & Macintyre, 2006; Cheng & Furnham, 2013; Clouston et al., 2015; Osler et al., 2008; Sjölund, Hemmingsson, & Allebeck, 2015). Second, drinking behaviours are known to be influenced by societal norms and attitudes towards drinking (Peele, 1997).

Finding higher IQ to be linked with a lower chance of being a current smoker is consistent with findings in previous studies (Osler et al., 2008; Wennerstad et al., 2010; Batty, Deary, Schoon, & Gale, 2006).The inverted u-shaped association between higher IQ and quantity of cigarettes smoked was novel to this study.

Two previous studies analysed the association between intelligence and oral health (Der et al., 2009; Sabbah & Sheiham, 2010). However, the study by Sabbah and Sheiham (2010) was cross sectional and neither study looked at the association with oral care habits. The findings of a positive association between IQ in youth and frequency of teeth brushing and flossing regularly in middle age, and the inverted u-shaped association with frequency of flossing are all novel to the present study.

The results observed in the present study that are inconsistent with the general findings in cognitive epidemiology (i.e. higher intelligence is linked with healthier behaviours and better health outcomes), which where the associations between higher intelligence and both skipping meals and snacking between meals as well as the u-shaped association between IQ and alcohol consumption and the inverted u-shaped associations between IQ and both number of meals skipped and cigarettes smoked, require further investigation. Future research could test for possible mediating factors that can help to explain these novel findings.

One possible explanation for the present findings suggests that adult SES mediates some of the association between intelligence in adolescence and health behaviours in middle age. One possible explanation for this is that individuals with a higher IQ tend to have access to higher status jobs and higher income (Cheng & Furnham, 2013; Herrnstein & Murray, 1994; Johnson, Gow, Corley, Starr, & Deary, 2010; Strenze, 2007), which results in more resources that could be invested into a healthier lifestyle. It is important to explain why education was not adjusted for as an indicator of adult SES in the present study. Education tends to correlate strongly with intelligence. For example Strenze (2007) conducted a meta analysis and found early life intelligence and educational attainment in adulthood correlated with *r* = 0.56 (*n* = 26,504), which suggests that much of the variability in educational attainment may be explained by variability in early life intelligence. It has also been suggested that education acts as a surrogate for aspects of early life intelligence because the two factors are so strongly correlated and share common genetic influences (Davies et al., 2016; Deary & Johnson, 2010; Hagenaars et al., 2016; Johnson, Deary, Silventoinen, Tynelius, & Rasmussen, 2010).

Another possible explanation for the present IQ-health behaviour associations is that more intelligent people are better equipped to pay attention to, internalise and respond to health promotion messages, which inform the public about healthy behaviours that help to prevent chronic health problems (Gottfredson & Deary, 2004; Taylor et al., 2003). Consistent with this is the definition of intelligence as the ability to learn, reason, solve novel problems, and think abstractly (Gottfredson, 1997; Gottfredson & Deary, 2004). Managing one's own health and preventing disease is a complex task that depends upon these cognitive abilities (Gottfredson & Deary, 2004). The non-linear associations between IQ and both exercise and flossing frequency may highlight that more intelligent people are most likely to do moderate amounts of these behaviours, which are linked with well-known health benefits (American Dental Association, 2016; Candow & Burke, 2007; O'Keefe et al., 2012; U.S Department of Health and Human Services, 2008; Wen et al., 2011; Wernbom et al., 2007). Engaging in high amounts of physical activity is thought to be linked with no clear additional health benefits and/or health damaging consequences (Lee, Morrison, Isserow, Heilbron, & Krahn, 2016; Seidl & Asplund, 2014; Candow & Burke, 2007; Wernbom et al., 2007). Engaging in exessive amounts of oral hygiene behaviours can lead to tooth damage and can be a sign of obsessive-compulsive disorder (Arora, Shahi, Sharma, Philip, & Pal Singh, 2013).

The few cases where higher IQ was linked with either unhealthy behaviours or behaviours that aren't clearly linked with better health were inconsistent with the explanation previously discussed. These outcomes were the U-shaped association between IQ and quantity of alcohol consumed, the inverted U-shaped association between IQ and number of cigarettes smoked, and the inverted U-shaped association between IQ and number of meals skipped, which were not explained by adult SES. More research is needed to help identify possible factors that help to mediate these relationships.

In addition to looking more closely at behaviours that are linked with non-communicable diseases, future research in cognitive epidemiology could also focus on the associations between IQ and behaviours associated with infectious disease such as HIV. Poor health literacy, regarding HIV, has been cited as one of the possible factors that contributes to the spread of the disease in sub-Saharan Africa (Oesterdiekhoff & Rindermann, 2007). Higher levels of education have been liked with better adherence to HIV treatment (Goldman & Smith, 2002). There is evidence that health literacy is a sub-component of general intelligence and education is known to correlate strongly with and may be a partial proxy for intelligence (Hagenaars et al., 2016; Herrnstein & Murray, 1994; Reeve & Basalik, 2014; Murray, 1998; Strenze, 2007). Intelligence may therefore be a factor that influences the spread and management of HIV in the population.

### 3.3 Strength & weaknesses

This study has a number of strengths. First, there is almost a 30-year gap between the measure of intelligence in youth and the measured health behaviours in middle age, so it is unlikely that the measure of intelligence was influenced by poor health or poor health habits in middle age. Second, the study was based on a large and representative sample of middle age American men and women (Bureau of Labor Statistics, 2012. Third, a greater breadth and more detailed measures of health behaviours were included in the present study than have been included in previous studies (Batty, Deary, & Macintyre, 2007; Batty, Deary, Schoon, & Gale, 2006; Clouston et al., 2015; Daly & Egan, 2017; Modig & Bergman, 2012; Sjölund et al., 2011; Sjölund, Hemmingsson, Gustafsson, & Allebeck, 2015; Taylor et al., 2003; Wennerstad et al., 2010). A fourth strength was the inclusion of self-reported measures of physical activity, dietary habits, drinking and smoking behaviours that have good criterion validity (Craig et al., 2003; Sabia et al., 2013; Sallis & Saelens, 2000; Cade, Burley, Warm, Thompson, & Margetts, 2004; Del Boca & Darkes, 2003; Patrick et al., 1994). A fifth strength was that the questions that measured oral health care were consistent with the WHO's recommendations for self-reported questions in surveys on oral health risk factors (World Health Organization, 2013).

This study had some limitations. First, there are a high number of missing data for total net family income. Because of this, the number of observations that made up the analytical samples was reduced; however the numbers were still large. Second, due to the socially sensitive nature of the questions on health behaviours, these questions are vulnerable to socially desirable responses where people will overestimate social desirable behaviours and underestimate socially undesirable behaviours (van de Mortel et al., 2008; Davis et al., 2010; Klesges et al., 2004; Motl, McAuley, & DiStefano, 2005; Dumitrescu, Kawamura, Toma, & Lascu, 2007; Tooze et al., 2004). This tends to decrease the validity of the scale and increase error in measurement. It was not possible to adjust for social desirability bias in the present study because the NLSY-79 2012 survey did not include a social desirability scale. Third, the responses to the two questions about reading nutritional labels were based on a likert scale. People who score lower on mental tests are more likely to have an extreme response style, which is the tendency to select response at one of the end-points of the scale (i.e. always or never) rather than selecting moderate responses (i.e. often, sometimes, or rarely) (Batchelor & Miao, 2016). This could have biased the present results.

## 4 Conclusion

The present study found evidence of links between higher IQ and a number of more favourable health related habits (i.e. engaging in physical activity, nutritional literacy, and oral hygiene habits, as well as not smoking, binge dinking, or consuming sugary drinks), which were largely independent of childhood SES, and often survived adjustment for adult socio-economic position. These findings, support the notion that certain health behaviours may lie on a pathway that links intelligence in early life with various health outcomes in adulthood. The few studies that have adjusted for health behaviours as a potential mediator found little to no attenuation of the estimated effect size of IQ on mortality (Batty, Deary, & Macintyre, 2006; Kuh, Richards, et al., 2004; Jokela, Batty, Deary, Gale, & Kivimäki, 2009. This might be because the majority of these studies are based on relatively young cohorts who have only been followed up into middle age. It is possible that health behaviours could mediate associations between IQ in youth and morbidity and mortality in old age. As the cohorts age, future studies could adjust for a range of health behaviours as a potential mediator of the IQ-morbidity/mortality association. In contrast to finding higher IQ to be linked with healthier behaviours, a few associations were identified between intelligence and behaviours that may or may not be linked with poor health (i.e. skipping meals & snacking between meals) and with behaviours that are known to be linked with poor health (i.e. drinking alcohol and the number of cigarettes smoked per day). More research into these findings, which are novel to this study, is required.

## Appendix A Supplementary data

Supplementary material Image 1
