# Supplementary material for: Intelligence in youth and health behaviours in middle age
Source: Intelligence. 2018 Jul-Aug;69:71–86. doi: 10.1016/j.intell.2018.04.005 (PMC6075942; doi:10.1016/j.intell.2018.04.005)
Supplement: Supplementary file 1 — Supplementary material [file mmc1.docx]

Table S1

Characteristics of the participants’ AFQT scores, childhood SES, and adult SES by behavioural outcome categories

|  |  | AFQT | | | Adult SES | | | Childhood SES | | |
| --- | --- | --- | --- | --- | --- | --- | --- | --- | --- | --- |
|  |  | obs | Mean (sd) | P value | obs | Mean (sd) | P value | obs | Mean (sd) | P value |
| **Are you able to do the following activities?** |  |  |  |  |  |  |  |  |  |  |
|  |  |  |  |  |  |  |  |  |  |  |
| Strength training activities | Yes | 6,163 | -0.31 (1.02) | p<.001 | 5,013 | 0.09 (0.81) | p<.001 | 6,163 | -0.41 (0.08) | p<.001 |
|  | No | 348 | -0.77 (0.91) |  | 226 | -0.29 (0.76) |  | 348 | -0.60 (0.98) |  |
|  |  |  |  |  |  |  |  |  |  |  |
| Moderate cardiovascular activities | Yes | 6,582 | -0.31 (1.02) | p<.001 | 5,348 | 0.08 (0.81) | p<.001 | 6,582 | -0.35 (1.08) | p<.001 |
|  | No | 163 | -0.83 (0.89) |  | 106 | -0.38 (0.80) |  | 163 | -0.67 (1.03) |  |
|  |  |  |  |  |  |  |  |  |  |  |
| Vigorous cardiovascular activities | Yes | 6,398 | -0.31(1.01) | p<.001 | 5,214 | 0.09 (0.81) | p<.001 | 6,398 | -0.34 (1.08) | p<.001 |
|  | No | 316 | -0.80 (0.92) |  | 201 | -0.40 (0.76) |  | 316 | -0.61 (1.01) |  |
| **How much of the following activities do you do?** |  |  |  |  |  |  |  |  |  |  |
|  |  |  |  |  |  |  |  |  |  |  |
| Strength training  (sessions/week) | 0 | 3,363 | -0.39 (1.01) | p<.001 | 2,691 | -0.04 (0.79) | p<.001 | 3,363 | -0.43 (1.07) | p<.001 |
|  | 1 to 3 | 1,711 | -0.08 (1.01) |  | 1,443 | 0.31 (0.77) |  | 1,711 | -0.16 (1.10) |  |
|  | ≥4 | 981 | -0.36 (1.02) |  | 794 | 0.14 (0.85) |  | 981 | -0.33 (1.09) |  |
|  |  |  |  |  |  |  |  |  |  |  |
| Moderate cardio vascular activity (minutes/week) | <75 | 2,787 | -0.35 (1.03) | p<.001 | 2,262 | 0.11 (0.83) | p<.001 | 2,787 | -0.40 (1.08) | p<.001 |
|  | $\geq$75 to 149 | 1,136 | -0.13 (1.06) |  | 933 | 0.21 (0.81) |  | 1,136 | -0.21 (1.09) |  |
|  | $\geq$150 to 509 | 1,580 | -0.26 (1.02) |  | 1,288 | 0.09 (0.81) |  | 1,580 | -0.29 (1.11) |  |
|  | $\geq$510 | 749 | -0.50 (0.90) |  | 602 | -0.18 (0.71) |  | 749 | -0.45 (0.99) |  |
|  |  |  |  |  |  |  |  |  |  |  |
| Vigorous cardiovascular activity (minutes/week) | <38 | 2,164 | -0.42 (1.02) | p<.001 | 1,710 | -0.05 (0.80) | p<.001 | 2,164 | -0.46 (1.09) | p<.001 |
|  | $\geq$38 to 74 | 698 | -0.16 (1.00) |  | 583 | 0.16 (0.79) |  | 698 | -0.26 (1.03) |  |
|  | $\leq$75 to 254 | 2,009 | -0.11 (1.00) |  | 1,715 | 0.27 (0.79) |  | 2,009 | -0.19 (1.08) |  |
|  | $\geq$255 | 1,258 | -0.41 (0.96) |  | 1,002 | 0.07 (0.81) |  | 1,258 | -0.36 (1.09) |  |
| **In the past week have you:** |  |  |  |  |  |  |  |  |  |  |
| Eaten fast food | Yes | 4,095 | -0.36 (1.00) | p=.010 | 3,341 | 0.05 (0.79) | p=.338 | 4,095 | -0.43 (1.06) | p<.001 |
|  | No | 2,892 | -0.30 (1.04) |  | 2,278 | 0.08 (0.85) |  | 2,892 | -0.27 (1.10) |  |
|  |  |  |  |  |  |  |  |  |  |  |
| Skipped any meals | Yes | 4,257 | -0.30 (1.00) | p<.001 | 3,435 | 0.06 (0.81) | p=.413 | 2,708 | -0.33 (1.07) | p=.004 |
|  | No | 2,708 | -0.40 (1.05) |  | 2,171 | 0.08 (0.83) |  | 4,257 | -0.41 (1.09) |  |
|  |  |  |  |  |  |  |  |  |  |  |
| Snacked between meals | Yes | 5,888 | -0.29 (1.02) | p<.001 | 4,781 | 0.09 (0.81) | p<.001 | 5,888 | -0.34 (1.08) | p=.002 |
|  | No | 1,073 | -0.56 (0.99) |  | 829 | -0.07 (0.81) |  | 1,073 | -0.45 (1.08) |  |
|  |  |  |  |  |  |  |  |  |  |  |
| Had any sugary drinks | Yes | 3,695 | -0.52 (0.98) | p<.001 | 2,925 | -0.08 (0.78) | p<.001 | 3,695 | -0.56 (1.03) | p<.001 |
|  | No | 3,286 | -0.13 (1.03) |  | 2,689 | 0.22 (0.81) |  | 3,286 | -0.15 (1.09) |  |
| **When shopping do you:** |  |  |  |  |  |  |  |  |  |  |
| Read nutritional information | Always | 2,061 | -0.21 (1.01) | p<.001 | 1,663 | 0.17 (0.82) | p<.001 | 2,061 | -0.24 (1.06) | p<.001 |
|  | Often | 1,465 | 0.03 (1.00) |  | 1,234 | 0.27 (0.80) |  | 1,465 | -0.12 (1.08) |  |
|  | Sometimes | 1,471 | -0.52 (1.02) |  | 1,160 | -0.05 (0.79) |  | 1,471 | -0.54 (1.07) |  |
|  | Rarely | 693 | -0.35 (0.98) |  | 562 | 0.01 (0.81) |  | 693 | -0.38 (1.07) |  |
|  | Never | 1,256 | -0.73 (0.90) |  | 967 | -0.21 (0.76) |  | 1,256 | -0.63 (1.03) |  |
|  |  |  |  |  |  |  |  |  |  |  |
| Read the ingredients | Always | 1,801 | -0.32 (1.03) | p<.001 | 1,416 | 0.07 (0.84) | p<.001 | 1,801 | -0.29 (1.07) | p<.001 |
|  | Often | 1,238 | -0.05 (0.99) |  | 1,037 | 0.24 (0.81) |  | 1,238 | -0.20 (1.09) |  |
|  | Sometimes | 1,663 | -0.35 (1.07) |  | 1,338 | 0.05 (0.81) |  | 1,663 | -0.41 (1.11) |  |
|  | Rarely | 829 | -0.19 (0.96) |  | 688 | 0.16 (0.78) |  | 829 | -0.29 (1.04) |  |
|  | Never | 1,417 | -0.67 (0.91) |  | 1,107 | -0.14 (0.76) |  | 1,417 | -0.59 (1.04) |  |
| **Smoking & Drinking** |  |  |  |  |  |  |  |  |  |  |
| Did you drink alcohol in the past month? | Yes | 3,830 | -0.10 (1.01) | p<.001 | 2,360 | 0.22 (0.79) | p<.001 | 3,830 | -0.15 (1.07) | p<.001 |
|  | No | 3,131 | -0.61 (0.96) |  | 3,250 | -0.15 (0.79) |  | 3,131 | -0.62 (1.04) |  |
|  |  |  |  |  |  |  |  |  |  |  |
| Did you have 6 or more drinks on one occasion in the past month? | Yes | 991 | -0.34 (1.00) | p<.001 | 827 | 0.02 (0.80) | p<.001 | 991 | -0.34 (1.05) | p<.001 |
|  | No | 2,832 | -0.02(1.00) |  | 2,420 | 0.29 (0.78) |  | 2,832 | -0.08 (1.07) |  |
|  |  |  |  |  |  |  |  |  |  |  |
| Do you smoke? | Yes | 1,733 | -0.63 (0.90) | p<.001 | 1,323 | -0.32 (0.76) | p<.001 | 1,733 | -0.51 (0.95) | p<.001 |
|  | No | 5,220 | -0.24 (1.04) |  | 4,266 | 0.18 (0.79) |  | 5,220 | -0.31 (1.12) |  |
| **Oral care** |  |  |  |  |  |  |  |  |  |  |
| Do you floss? | Yes | 5,085 | -0.22 (1.00) | p<.001 | 4,198 | 0.16 (0.79) | p<.001 | 5,085 | -0.29 (1.09) | p<.001 |
|  | No | 1,505 | -0.56 (1.01) |  | 1,151 | -0.17 (0.84) |  | 1,505 | -0.49 (1.05) |  |
|  |  |  |  |  |  |  |  |  |  |  |
| How often do you floss (times per week)? | Never | 1,506 | -0.55 (1.01) | p<.001 | 1,151 | -0.17 (0.84) | p<.001 | 1,506 | -0.49 (1.05) | p<.001 |
|  | 1 to 4 | 1,825 | -0.16 (1.01) |  | 1,538 | 0.15 (0.77) |  | 1,825 | -0.25 (1.08) |  |
|  | 5 to 7 | 2,160 | -0.16 (1.03) |  | 1,788 | 0.22 (0.79) |  | 2,160 | -0.23 (1.08) |  |
|  | > 7 | 1,100 | -0.43 (0.91) |  | 872 | 0.06 (0.78) |  | 1,100 | -0.45 (1.10) |  |
|  |  |  |  |  |  |  |  |  |  |  |
| How many times a day do you brush your teeth? | < once | 188 | -0.78 (1.10) | p<.001 | 128 | -0.36 (0.91) | p<.001 | 188 | -0.70 (1.11) | p<.001 |
|  | 1 to <2 | 1,570 | -0.38 (1.04) |  | 1,260 | -0.04 (0.81) |  | 1,570 | -0.41 (1.02) |  |
|  | 2 + | 4,845 | -0.25 (1.00) |  | 3,970 | 0.15 (0.80) |  | 4,845 | -0.29 (1.09) |  |
